# Exosomes in Inflammatory Bowel Disease: Mechanisms, Diagnostic Potential, and Engineering Strategies for Precision Therapy

**DOI:** 10.7150/ijms.131676

**Published:** 2026-05-01

**Authors:** Zhong-kuo Zhao, Jia-le Ma, Fei Lu, Shou-jie Wang

**Affiliations:** 1 Department of Gastrointestinal Surgery, the Fourth Affiliated Hospital, Zhejiang University School of Medicine, Yiwu 322000, China.; 2 Department of Plastic Surgery, the Fourth Affiliated Hospital, Zhejiang University School of Medicine, Yiwu 322000, China.; 3 Department of Plastic Surgery, The First Affiliated Hospital, Zhejiang University School of Medicine, Hangzhou 310003, Zhejiang, China.

**Keywords:** inflammatory bowel disease, engineered exosomes, precision therapy, immune modulation

## Abstract

Inflammatory bowel disease (IBD) is a chronic gastrointestinal disorder characterized by immune dysregulation, epithelial barrier dysfunction, and microbial imbalance. Despite progress in biologic therapies, challenges such as variable efficacy, systemic side effects, and the lack of reliable biomarkers remain significant obstacles in clinical management. Exosomes, key mediators of intercellular communication, play a pivotal role in IBD's pathogenesis by transporting bioactive substances. Increasing evidence links exosomes to critical IBD processes, including Th17/Treg imbalance, inflammasome activation, and host-microbiome interactions. Exosomes also show potential as minimally invasive biomarkers for disease activity and subtype differentiation. Furthermore, advancements in exosome engineering, including surface modification and hybrid nanostructure development, enhance their potential for targeted drug delivery and immune modulation in IBD. This review summarizes the role of exosomes in IBD, their diagnostic potential, and emerging exosome-based therapeutic strategies.

## 1. Introduction

Inflammatory bowel disease (IBD) is a chronic immune-mediated inflammatory disorder that primarily affects the gastrointestinal tract, including ulcerative colitis (UC), Crohn's disease (CD), and indeterminate IBD^1^.

Over the past few decades, the number of IBD patients has surpassed 5 million, and its global incidence has been steadily increasing, particularly in Asia and developing countries^2^. Young and middle-aged patients often face severe complications, which not only cause significant distress to individuals but also place a substantial economic burden on families and society. This issue has garnered widespread attention in the field of biomedical research^3^.

The pathogenesis of IBD results from a multifactorial interaction involving genetic susceptibility, immune dysregulation, environmental factors, and imbalances in the gut microbiota^4^. Notably, CD exhibits certain distinctive pathological features that further reflect the complexity of disease mechanisms. One such feature is creeping fat (CrF), characterized by mesenteric adipose tissue hypertrophy and its “wrapping” around inflamed intestinal segments. This phenomenon is closely associated with transmural inflammation, fibrostenosis, and disease progression, and is largely absent in UC^5^. Emerging evidence suggests that CrF is not merely a passive structural change but an active immunometabolic compartment that participates in intestinal inflammation through intercellular communication, including extracellular vesicle (EV)-mediated signaling. Recent studies indicate that EVs derived from creeping fat-associated adipose-derived stem cells (CrF-ADSCs) may exert context-dependent effects in CD. For example, CrF-derived exosomes enriched in miR-103a-3p have been reported to promote intestinal fibrosis via TGFBR3/Smad2/3 signaling^6^, whereas other studies suggest that CrF-ADSC-derived EVs containing miR-132-3p may attenuate inflammation by enhancing lymphangiogenesis and lymphatic drainage^7^. These findings highlight the dual and context-dependent roles of tissue-specific EVs in CD and further underscore the importance of EV-mediated communication in the mesenteric-intestinal axis. At present, there is no cure for IBD, and surgical intervention remains a last resort in its treatment^8^. The primary aim of pharmacological therapy is to achieve long-term remission, thereby minimizing the need for surgery^9^. Although current treatments, such as aminosalicylates, corticosteroids, immunosuppressants, and biologic agents, can alleviate inflammation, many patients exhibit poor responses to existing therapies, and the long-term side effects of these treatments are concerning^10^-^13^. Therefore, there is an urgent need to explore more targeted, safer, and lower-side-effect therapeutic strategies.

Extracellular vesicles (EVs) are membrane-bound nanoparticles secreted by most cell types and function as important mediators of intercellular communication. EVs represent a heterogeneous population of vesicles that can be broadly classified based on their biogenesis and size, including exosomes (approximately 30-150 nm), microvesicles (100-1000 nm), and apoptotic bodies (>1000 nm)^14^. Among these subtypes, exosomes originate from the endosomal pathway and are released when multivesicular bodies fuse with the plasma membrane. These vesicles carry a wide range of bioactive cargos, including nucleic acids, proteins, lipids, and metabolites, enabling them to modulate cellular signaling and influence various physiological and pathological processes^15^, ^16^. In addition to mammalian cell-derived vesicles, recent studies have identified plant-derived exosome-like nanoparticles (PDENs), which are nanoscale vesicles isolated from edible plants such as ginger, grape, and tea leaves. Although PDENs share several structural and functional similarities with mammalian exosomes—including a lipid bilayer membrane and the ability to transport bioactive cargos—their biogenesis pathways remain less clearly defined. Increasing evidence suggests that EVs, including exosomes and PDENs, participate in key processes involved in IBD^17^, such as immune regulation, epithelial barrier maintenance, and host-microbiota interactions. In this review, the term EVs is used as a general umbrella term when referring to extracellular vesicles collectively, while exosomes specifically refer to vesicles of endosomal origin. PDENs are discussed separately to highlight their distinct biological characteristics and emerging applications in intestinal disease therapy and oral drug delivery. This review provides a comprehensive overview of the role of exosomes in IBD, with a focus on their mechanistic involvement in disease pathogenesis, their potential as diagnostic biomarkers, and the latest advancements in exosome-based engineering strategies for therapeutic applications. Additionally, we discuss the current challenges and future prospects related to exosome standardization, large-scale production, and clinical translation, emphasizing their potential as a multifunctional precision medicine platform.

## 2. Exosome-Based Therapy for IBD: Main Mechanisms

Immune dysregulation plays a pivotal role in the pathogenesis of IBD. In IBD patients, the expression levels of pro-inflammatory cytokines such as TNF-α, IL-1β, and IL-6 are significantly elevated, continuously driving chronic intestinal inflammation^18^. Additionally, the balance between T helper 17 (Th17) cells and T regulatory (Treg) cells is crucial. Th17 cells primarily secrete pro-inflammatory cytokines such as IL-17, IL-21, and IL-22, which are involved in intestinal immune responses, while Treg cells maintain immune tolerance by secreting immunosuppressive cytokines such as IL-10^19^. In IBD patients, enhanced Th17 cells function and impaired or reduced Treg cells function or numbers lead to immune imbalance, exacerbating intestinal inflammation^20^, ^21^. Recent studies have also highlighted the importance of ubiquitination-mediated regulation in NF-κB signaling during intestinal inflammation. For example, Jiang *et al*. reported that N4BP3 promotes K48-linked ubiquitination of IκBα, leading to its proteasomal degradation and subsequent activation of the TLR4-NF-κB signaling pathway, thereby exacerbating inflammatory responses in inflammatory bowel disease^22^. Therefore, anti-inflammatory and immunomodulatory therapies are central to IBD treatment. Exosomes can regulate immune cell function by carrying miRNAs, cytokines, and lipid mediators. For example, Yang *et al*. discovered that exosomes derived from M2b macrophages increased the number of Treg cells and IL-4 levels in the spleens of colitis mice, while inhibiting key cytokines associated with colitis (such as IL-1β, IL-6, and IL-17A), effectively alleviating DSS-induced colitis in mice^23^. Furthermore, exosomes derived from mesenchymal stem cells (MSC-Exos) contain miR-146a, which targets the TRAF6/IRAK1 axis, reducing the release of IL-1β and TNF-α, thereby mitigating the inflammatory response^24^. In summary, current evidence suggests that exosomes, by carrying miRNAs, proteins, long non-coding RNAs, and other signaling molecules, can modulate the inflammatory microenvironment, inhibit pro-inflammatory pathways, enhance regulatory immunity, and thereby help correct Th17/Treg imbalance, reduce intestinal inflammation, and promote mucosal healing. Similarly, Lv *et al*. demonstrated that macrophage-derived exosomes regulate immune cell function and balance pro-inflammatory and anti-inflammatory responses, thereby promoting tissue repair. This highlights the potential of exosomes as therapeutic agents in controlling chronic inflammation and immune dysregulation, offering valuable insights into their potential application in the treatment of IBD^25^.

Pyroptosis of intestinal epithelial cells (IECs) is a critical factor in the progression of IBD. Moderate pyroptosis activation can enhance intestinal immune defense, whereas excessive inflammasome activation triggers an inflammatory cascade, leading to increased intestinal tissue damage^26^, ^27^. Pathogens or injury signals excessively activate the NLRP3 inflammasome, prompting Caspase-1 to cleave Gasdermin D (GSDMD), forming membrane pores, which causes epithelial cell lysis and the release of pro-inflammatory cytokines such as IL-1β and IL-18, thus amplifying the intestinal inflammatory response^28^. Targeting various aspects of pyroptosis can significantly reduce inflammation and intestinal mucosal damage, and inhibiting the NLRP3 pathway holds considerable therapeutic potential, possibly offering a new strategy for IBD treatment^29^. For example, exosomes derived from bone marrow mesenchymal stem cells (BMSCs-Exo) contain TSG-6, which plays a key role in alleviating DSS-induced IBD by inhibiting pyroptosis and upregulating tight junction proteins, thereby providing dual protection to the intestinal mucosa. TSG-6-enriched BMSCs-Exo significantly alleviated pyroptosis and intestinal inflammation in a mouse IBD model by inhibiting NLRP3 inflammasome activation and upregulating tight junction proteins^30^. Another study demonstrated that exosomes derived from human umbilical mesenchymal stem cells (hucMSCs-Exo) activated the SIRT1-FXR pathway, inhibiting NLRP3 inflammasome activation, alleviating intestinal inflammation, and repairing the intestinal barrier function, showcasing their potential for IBD treatment^31^. In summary, exosomes offer a novel cell-free therapeutic approach for IBD intervention by inhibiting IEC pyroptosis, restoring barrier integrity, and modulating the local immune microenvironment. Dysbiosis has been widely recognized as one of the core factors in the pathogenesis and progression of IBD^32^. Under healthy conditions, the gut microbiota maintains high diversity, with dominant species (such as *Faecalibacterium prausnitzii*, *Roseburia*, and certain lactobacilli) producing short-chain fatty acids (SCFAs), indole derivatives, and other metabolites. These substances act on epithelial and immune cells to maintain barrier homeostasis and mucosal immune tolerance^33^-^35^. However, when dysbiosis occurs, characterized by a decrease in beneficial bacteria and an overgrowth of harmful bacteria (such as Proteobacteria and Escherichia-Shigella), as well as disruption of the microbiota network structure, it can trigger a cascade of events. Increased intestinal barrier permeability facilitates the entry of bacteria or their products into the mucosal layer. These invading microbes and their metabolites (such as toxic lipopolysaccharides, secondary bile acids, and oxidants) activate innate immunity, including the NLRP3 inflammasome, and adaptive immune responses, particularly the expansion of Th17 cells. Ultimately, this creates a pro-inflammatory environment that accelerates the onset, relapse, or exacerbation of IBD^34^, ^36^. Recent studies have also highlighted the importance of microbiota in maintaining intestinal barrier integrity and regulating immune responses. For example, Su *et al*. demonstrated that microbiota-derived metabolites can enhance intestinal barrier function by promoting tight junction protein expression and reducing epithelial cell permeability, thereby reinforcing the gut mucosal defense^37^. Similarly, Taj *et al*. found that microbiota modulation could restore epithelial barrier function and regulate immune cell activation, leading to a reduction in inflammatory cytokine production^38^. This underscores the therapeutic potential of microbiota modulation in IBD treatment. In this context, exosomes derived from beneficial microbiota emerge as promising therapeutic agents by modulating the gut microbiota and promoting intestinal barrier integrity. For example, exosomes derived from *Lactobacillus johnsonii* (L. johnsonii-derived EVs) exhibited significant protective effects in a DSS-induced colitis mouse model. Zhao *et al*. reported that these exosomes could reshape the gut microbiota, increasing beneficial bacteria such as Lactobacillus and *Clostridia*, while significantly reducing pathogenic bacteria like *Escherichia-Shigella*. Metabolomic analysis showed that these exosomes increased taurine levels in the gut, which helped regulate the Th17/Treg balance and inhibited the expression of pro-inflammatory cytokines (IL-6, IL-1β), thereby effectively alleviating inflammation^39^. Furthermore, dietary L. johnsonii-derived EVs (LJ-EVs) have been shown to be taken up by epithelial cells, activating the Nrf2/HO-1 antioxidant pathway to reduce oxidative stress damage, while also restoring microbiota diversity and functional metabolic networks, further improving colitis symptoms^40^. In summary, exosomes can modulate the gut microbiome through various mechanisms, promoting the proliferation of beneficial bacteria, inhibiting the expansion of harmful bacteria, restoring microbiota metabolic balance, and ultimately reducing mucosal inflammation and repairing the epithelial barrier.

Building upon the above mechanistic pathways, a more unified model of exosome action in IBD can be proposed by integrating vesicle origin, trafficking routes, and cell-type-specific uptake. Different exosome sources exhibit distinct biodistribution and targeting preferences. Systemically administered exosomes, such as those derived from mesenchymal stem cells or macrophages, primarily traffic through the circulation and mesenteric lymphatic system, accumulating in inflamed intestinal tissues where they are preferentially taken up by IECs and lamina propria macrophages^23^, ^30^, ^31^. In contrast, orally delivered vesicles—such as microbiota-derived or plant-derived exosome-like nanoparticles—largely remain within the gastrointestinal lumen, where they are directly internalized by IECs and resident phagocytes, exerting more localized effects^40^, ^41^. This spatially resolved delivery pattern suggests that IECs and macrophages act as primary “first responders,” while downstream adaptive immune modulation, including CD4⁺ T cell reprogramming toward a Treg-dominant phenotype, represents a secondary, amplification layer of regulation.

Despite the growing body of mechanistic insights, it is important to distinguish between causality and correlation in current exosome research. Many studies report associations between exosome cargo, including specific miRNAs or proteins, and improvements in inflammation, barrier function, or microbiota composition; however, direct causal evidence, including loss-of-function and cargo-specific manipulation experiments, remains relatively limited. For instance, while TSG-6 enrichment or NLRP3 inhibition is frequently linked to therapeutic benefit, definitive attribution of these effects to individual exosomal components often requires further validation using targeted depletion, genetic editing, or rescue experiments. Therefore, caution is warranted when interpreting exosome-mediated effects as strictly causal rather than correlative, particularly in complex, multi-cellular disease contexts like IBD.

In addition, the current evidence base is heavily skewed toward preclinical models. Most mechanistic studies—including those involving Th17/Treg modulation, NLRP3 inflammasome inhibition, and microbiome reshaping—are derived from DSS- or TNBS-induced colitis models in rodents. While these models provide valuable insights into inflammatory pathways and therapeutic potential, their translational relevance to human IBD remains incomplete. Clinical evidence is largely limited to observational studies of exosome-associated biomarkers in patient cohorts, with relatively few interventional trials evaluating therapeutic efficacy. As such, the strength of evidence can be broadly graded as high for mechanistic plausibility in animal models, moderate for biomarker associations in human samples, and currently low for confirmed clinical efficacy. Future studies integrating well-designed human trials with mechanistic validation will be essential to bridge this gap and establish exosomes as a truly evidence-based precision therapy platform in IBD.

**Figure 1:**
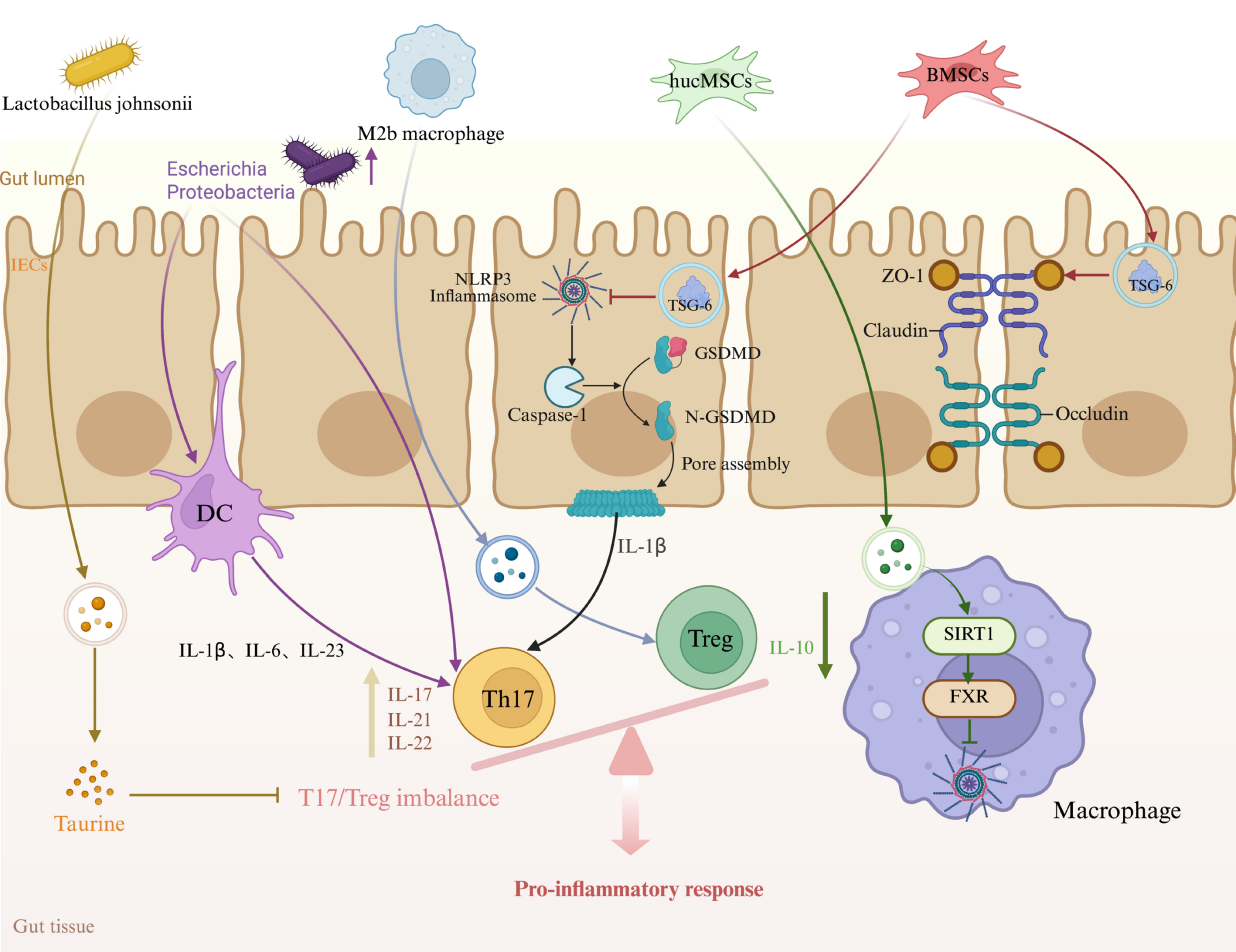
** Mechanisms of Exosome Action in Inflammatory Bowel Disease (IBD).** Exosomes, by carrying bioactive molecules such as miRNAs, proteins, and lipids, regulate the function of immune cells and intestinal epithelial cells. They are involved in immune modulation, pyroptosis, and intestinal barrier repair, playing a crucial role in the onset and progression of IBD. hucMSCs: human umbilical mesenchymal stem cells, BMSCs: bone marrow mesenchymal stem cells, Th17: T helper 17 cells, Treg: T regulatory cells, GSDMD: Gasdermin D, IECs: Intestinal epithelial cells.

In summary, immune dysregulation, intestinal epithelial cell pyroptosis, and gut microbiota dysbiosis collectively drive the onset and progression of IBD. Exosomes, by carrying miRNAs, proteins, and other bioactive molecules, can simultaneously regulate the Th17/Treg balance, inhibit NLRP3-mediated pyroptosis, and reshape the gut microbiota, thereby alleviating inflammation and repairing the epithelial barrier on multiple levels (Figure 1). Their multi-target, engineerable nature makes exosomes a highly promising cell-free therapeutic approach for precision medicine in IBD treatment.

## 3. Exosome-Based Diagnostic Applications in IBD

Traditional diagnosis of IBD relies on endoscopy and histopathology, which are invasive and difficult to perform dynamic monitoring^42^. Exosomes, due to their high stability and the ability to be detected in various bodily fluids such as blood, saliva, and feces, are considered important signal carriers for IBD liquid biopsy^43^. In current studies, exosomes are typically obtained from biological fluids or cell culture supernatants using ultracentrifugation, precipitation-based methods, size-exclusion chromatography, or immunoaffinity-based approaches. Their identification and validation generally require a combination of particle size and morphology assessment, together with the detection of canonical exosomal markers such as CD9, CD63, and CD81, in order to ensure the relative purity and biological relevance of the isolated vesicles^44^. This methodological basis has supported the rapid development of exosome-related diagnostic research in IBD. On one hand, numerous studies have confirmed disease-specific changes in exosomes from IBD patients, which can serve as a foundation for engineered diagnostics. For example, plasma exosomal lncRNA H19 can distinguish active and remission phases of IBD with an AUC > 0.95^45^. A five-miRNA signature (miR-1246, miR-142-3p, miR-16-5p, miR-301a-3p, miR-4516) in salivary exosomes has been proposed as a non-invasive diagnostic biomarker for IBD^46^. Changes in the expression of small RNA in fecal exosomes may also reflect the degree of intestinal mucosal inflammation, providing potential for early diagnosis^47^. In summary, miRNA/lncRNA biomarkers from exosomes derived from various fluids (blood, tissue, and feces) offer a verifiable marker library for engineered diagnostics.

On the other hand, the concept of engineered exosomes as diagnostic carriers is gaining attention. Through cargo engineering, fluorescent/near-infrared probes, MRI contrast agents, and nanoenzymes can be loaded into exosomes for responsive imaging of the inflammatory microenvironment (such as ROS, proteases, TNF-α)^48^, ^49^. Surface engineering strategies, including coupling anti-MAdCAM-1 antibodies or α4β7 integrin-targeting ligands/homing peptides to exosome/nanocarrier surfaces, have demonstrated enhanced specificity for inflammation site enrichment in colitis mouse models. They can also be used for molecular imaging techniques, such as fluorescence or PET/CT, to localize active inflammation. This provides a technical foundation for subsequent non-invasive *in vivo* diagnostics and dynamic monitoring^50^, ^51^. Although the pure diagnostic application of engineered exosomes in IBD is still in its early stages, strategies for targeting inflammatory sites and releasing readable signals show the potential for disease localization by the carriers themselves. In comparison, engineered exosome detection platforms exhibit greater advantages in clinical translatability. Recently, various novel biosensing technologies have been integrated into exosome analysis systems, including magnetic bead enrichment combined with electrochemical sensing, optical detection based on surface plasmon resonance or surface-enhanced Raman scattering (SPR/SERS), lateral flow assay (LFA) platforms, and upconversion nanoparticle-mediated LRET wash-free sensors. These methods have achieved high sensitivity, rapid response, and low sample consumption detection of exosomes and the miRNAs they carry, while maintaining good stability and reproducibility in complex biological samples^44^, ^52^. Based on this, simply replacing the general recognition unit with IBD-related specific probes (such as lncRNA H19 or specific miRNA panels) can construct an integrated "exosome-engineered sensor" diagnostic system, providing a feasible path for translation into point-of-care testing (POCT) scenarios. However, several constraints must be addressed to translate exosome-based diagnostics into clinical practice. First, the methods for exosome isolation lack full standardization, with considerable variability between platforms and protocols^53^. In addition, pre-analytic factors, such as sample collection, handling, and storage, can significantly affect exosome detection, leading to inconsistent results across studies^54^. Secondly, confounding inflammation sources, such as infections or other chronic conditions, can affect the expression of exosome-derived biomarkers and lead to potential diagnostic errors. Moreover, cohort size and subtype separation remain critical factors in establishing robust diagnostic models. Larger and more diverse patient cohorts are needed to validate the accuracy of exosome-based biomarkers, and the heterogeneity of IBD subtypes must be considered to ensure diagnostic specificity. Finally, ensuring reproducibility across platforms is a major challenge, as different laboratories and diagnostic platforms may produce inconsistent results. Addressing these challenges will be essential to realize the potential of engineered exosomes for IBD diagnostics.

Overall, the research paradigm of engineered exosomes in IBD diagnosis is shifting from the early exploration phase centered on "single biomarker screening" to a more systematic development phase characterized by "carrier engineering, signal amplification strategies, and deep integration with detection platforms." Future research will focus on several key aspects: firstly, the development of exosome diagnostic models that integrate multi-omics data, including miRNAs, lncRNAs, proteins, and lipids, to improve the accuracy of distinguishing IBD subtypes and assessing disease activity; secondly, joint analysis of exosomes from multiple sources, such as blood, saliva, and feces, to more comprehensively reflect systemic inflammation and local pathological features of the intestine; and thirdly, the development of microfluidic integrated detection platforms that combine separation, enrichment, and detection functions, further simplifying the operational workflow and lowering clinical application barriers. As the standardization of exosome isolation technology and the ongoing bio-safety assessment of engineered modifications continue to progress, the clinical translational potential of exosomes in IBD diagnosis is becoming increasingly prominent, and they are expected to evolve into a new generation of precise diagnostic tools.

## 4. Recent Strategies in Engineered Exosome-Based Therapy for IBD

With the continuous advancement of biologic therapies, natural exosomes have demonstrated good safety and therapeutic potential in various disease models. However, their clinical translation, particularly in the treatment of complex diseases such as IBD, still faces several key challenges. First, the heterogeneity of the molecular cargo in natural exosomes leads to uncertain therapeutic outcomes and weak targeting ability. Second, most exosomes are cleared by the reticuloendothelial system after systemic administration, making precise targeting of intestinal mucosal lesions difficult. Additionally, exosome production is relatively low, with batch-to-batch variability, and their short *in vivo* half-life affects stability and dose control^55^, ^56^. To overcome these limitations, engineered exosomes have emerged as a promising solution. This review focuses on summarizing various engineering approaches, including gene editing of parent cells, surface functionalization of exosomes, targeted loading of therapeutic cargos, and exosome-nano structure hybridization, to enhance the functionality of exosomes at the source level, thereby improving their application value and therapeutic efficacy in the treatment of IBD (Table 1).

### 4.1 Engineering of exosome-producing cells

Genetic modification and optimization of culture conditions in exosome-producing cells can enhance the therapeutic efficacy and targeting capability of the resulting exosomes. On one hand, gene editing technologies are used to increase the content of specific therapeutic factors within exosomes, such as overexpressing anti-inflammatory genes or silencing pro-inflammatory genes. Wu *et al*. found that knockdown of the anti-inflammatory molecule TSG-6 in MSCs resulted in exosomes that lost their ability to alleviate colitis in mice. In contrast, exosomes derived from normal MSCs, which are rich in TSG-6, significantly inhibited NLRP3 inflammasome activation and reduced pyroptosis in colonic epithelial cells^30^. Conversely, increasing NLRP3 expression in MSCs promotes the production of the anti-inflammatory cytokine IL-10 in exosomes, thereby enhancing their therapeutic effect on colitis^57^. On the other hand, metabolic and microenvironmental preconditioning are also effective strategies. Hypoxic preconditioning can alter the metabolic state of parent cells, leading to exosomes that are enriched with specific factors, such as microRNAs, which enhance tissue repair and anti-inflammatory properties. For example, exosomes derived from hair follicle MSCs cultured under hypoxic conditions (Hy-Exos) demonstrated stronger therapeutic effects in a colitis model. Li *et al*. reported that Hy-Exos are rich in miR-214-3p, which promotes mitochondrial autophagy by inhibiting the PI3K/AKT/mTOR pathway, thereby alleviating oxidative damage and inflammation in mice with ulcerative colitis.^58^. Additionally, exosomes secreted by cells pretreated with inflammatory cytokines (such as LPS or cytokine stimulation) are more efficient in immune modulation. These strategies hold promise for enhancing the therapeutic potential of exosomes in IBD treatment.

Overall, genetic regulation and microenvironmental preconditioning of exosome-producing cells enable precise modulation of exosome cargo composition, significantly enhancing their anti-inflammatory and immunoregulatory effects in the treatment of inflammatory bowel disease.

### 4.2 Drug loading and cargo engineering of exosomes

To meet the therapeutic needs of "local high exposure in the intestine, controlled systemic exposure" for IBD, researchers have progressively developed exosomes and plant-derived exosome-like nanoparticles (PDENs) as oral delivery platforms, conducting systematic research on their drug loading and delivery performance^59^. Benefiting from their nanoscale structure, biological membrane composition, and relative tolerance to the gastrointestinal environment, exosomes can partially protect the therapeutic factors they carry from digestive degradation and promote their local enrichment in the intestine, especially in the inflamed colonic regions^60^. Current studies indicate that exosomes and PDENs can be used to load a variety of therapeutic molecules, including small molecule drugs, natural products, and nucleic acid-based factors. These systems have shown enhanced local efficacy and improved safety profiles in IBD animal models^61^. Exogenous loading is typically carried out after vesicle isolation and purification, and includes passive incubation/intercalation based on hydrophobic interactions, as well as physical permeation techniques such as electroporation, ultrasound, freeze-thaw cycles, and extrusion, which improve encapsulation efficiency of small molecules, nucleic acids, or protein factors and enhance their stability in the gastrointestinal environment^62^. For example, methotrexate (MTX), loaded into grapefruit-derived nanovesicles, achieves relative enrichment and controlled release at the colon site, reducing systemic toxicity and enhancing local anti-inflammatory effects in animal models of colitis^63^. Ginger-derived nanovesicles (GDNVs) loaded with hydrophobic natural products such as curcumin have also been used to enhance local drug exposure at the mucosa and improve the inflammatory microenvironment, with these strategies being actively pushed towards clinical translation^64^. In the case of nucleic acid drugs, exosome-based delivery of siRNA or microRNA has become a significant research direction. For instance, loading anti-TNF-α siRNA into milk-derived exosomes and administering it orally helps resist gastrointestinal degradation, promotes deposition in the colon, and facilitates uptake by target cells, thereby downregulating the expression of pro-inflammatory factors like TNF-α and alleviating mucosal damage^65^.

Overall, the drug loading and delivery engineering of exosomes and PDENs significantly expands the range of therapeutic agents for IBD and offers new strategies for multi-target, low systemic exposure, and localized mucosal treatment. However, several key challenges remain, including balancing drug encapsulation efficiency and vesicle structural stability under different loading strategies, large-scale production, batch-to-batch consistency, and reproducibility of *in vivo* distribution and colonic accumulation^66^. Systematic resolution of these issues will be a critical prerequisite for advancing exosome-based drug delivery systems toward clinical application.

### 4.3 Chemical or physical modification of exosomes

Post-isolation engineering of exosomes is gradually becoming one of the core strategies to enhance their *in vivo* stability and targeted delivery efficiency^67^. Chemical modification typically utilizes reactive groups on the membrane surface, enabling controlled decoration with ligands, tracers, and functional polymers through bioorthogonal click chemistry (such as CuAAC and SPAAC) or mild covalent coupling^68^, ^69^. In contrast, physical modification relies more on hydrophobic lipid anchoring, membrane fusion, and polysaccharide/polymer coating or layer-by-layer (LBL) self-assembly to enhance structural stability, adhesion, and penetration properties in complex barrier environments like the gastrointestinal tract, while also modulating their tissue distribution profile^70^-^72^. In IBD-related delivery research, Kang *et al*. conjugated hyaluronic acid (HA) to Red cabbage-derived EVs (Rabex) using a lipid-polyethylene glycol bridging strategy, creating HA-engineered t-Rabex. The HA-CD44 recognition enhanced its uptake by intestinal epithelial and immune cells, promoting intestinal accumulation. In a DSS-induced colitis model, t-Rabex at a lower dose achieved superior therapeutic benefits compared to unmodified Rabex, suggesting that "surface ligation" can effectively amplify the local therapeutic exposure of PDENs^73^. Further, the LBL self-assembly of "nano-armors" offers enhanced barrier compatibility for oral delivery. Deng *et al*. used chitosan/hyaluronic acid to coat milk-derived exosomes via LBL, significantly improving their stability, controlled release, and mucosal delivery efficiency during gastrointestinal transit. In a DSS-induced UC model, even when the exosome dose was halved, the treatment still significantly improved the intestinal barrier (including physical, mucus, and immune barriers) and alleviated inflammatory phenotypes, demonstrating the additive effects of "shell stabilization and enhanced local retention"^74^. It is worth noting that surface engineering can synergistically enhance drug-loading strategies: for example, Jing *et al*. used the cell-penetrating peptide TAT to mediate protein loading, enabling milk-derived exosomes to simultaneously deliver anti-TNF-α nanobodies and antimicrobial peptides. Oral administration of these exosomes reduced local TNF-α levels and improved the pathological outcomes of both acute and chronic UC^75^.

Overall, post-isolation modification of exosomes through chemical and physical engineering methods significantly enhances their *in vivo* stability and targeted delivery capacity. Strategies like surface ligation and LBL coating can improve exosome stability, mucosal enrichment, and local retention in the gastrointestinal environment, achieving better therapeutic effects in IBD models at lower doses. Further combining these strategies with biomacromolecule-based drug delivery could enhance local therapeutic exposure in the colon and reduce off-target effects, providing a promising translational pathway for precise oral delivery of therapeutics in intestinal diseases.

### 4.4 Hybrid engineering of exosomes with nanomaterials

Hybrid engineering of exosomes and nanomaterials has emerged as a powerful paradigm for precision therapy in IBD, integrating the intrinsic biological functionality of endogenous vesicles with the tunable physicochemical properties of synthetic nanocarriers. By overcoming key limitations of conventional therapeutics—including poor stability in the inflamed gastrointestinal milieu, limited site-specific accumulation and off-target toxicity—this strategy establishes a versatile platform for long-term, safe and potentially personalized IBD treatment^76^. Within this framework, PDENs have evolved from naturally occurring delivery vehicles into deeply engineerable bio-synthetic hybrid systems. Their membrane architectures, encompassing distinctive lipid compositions, glycosylation patterns and membrane-associated proteins, function as programmable modules for mucosal compatibility and biological recognition. When integrated with organic or inorganic nanomaterials through core-shell coating, membrane fusion or reverse engineering strategies, PDEN-based hybrids exhibit coordinated enhancement in oral stability, mucus penetration, inflammatory-site retention, cargo capacity and stimulus-responsive release^77^, ^78^. As a result, PDENs are no longer passive carriers but integral components of multifunctional platforms that couple drug delivery with immunomodulation and tissue repair^79^.

Representative studies illustrate how the targeting functions of PDEN membranes can be rationally amplified through hybridization. Grape-derived exosome-like nanoparticles preferentially interact with intestinal stem cells and activate regenerative signaling pathways, highlighting the engineering value of membrane-encoded targeting motifs^80^. Building on this principle, PDEN membranes have been widely employed as biomimetic shells for synthetic nanoparticle cores, enabling biological navigation by surface glycans and proteins while delegating high drug-loading efficiency and responsive release to the engineered core^81^. Notably, reverse-engineering approaches inspired by the intrinsic colon tropism of ginger-derived vesicles have enabled the reconstruction of lipid nanoparticles with defined membrane compositions for oral, colon-specific delivery of IL-22 mRNA, exemplifying a translational route from natural vesicles to scalable and compositionally controlled nanomaterials^82^.

Beyond epithelial targeting, hybrid systems also exploit pre-organized glycan ligands on PDEN surfaces to amplify immune cell specificity. Tea-derived nanovesicles (NTs) enriched in galactose residues undergo receptor-mediated uptake by macrophages, allowing localized immunoregulation and barrier restoration within the inflamed mucosa^83^. From an engineering perspective, such naturally glycosylated membranes constitute a pre-assembled targeting layer that can be seamlessly integrated with polymeric coatings, ionic crosslinking or inorganic cores, thereby unifying immune modulation and multimodal drug delivery within a single mucosal therapeutic architecture^84^.

The therapeutic synergy achievable through hybridization is further exemplified by ginger-derived nanoparticles, which combine intrinsic bioactive cargos—including gingerols, shogaols and regulatory miRNAs—with engineered nanocarriers. When cloaked onto metal-organic framework nanoparticles such as ZIF-8, ginger exosome membranes confer colon tropism, macrophage affinity and gastric protection, while the synthetic core enables high siRNA loading and acid-responsive release. This cooperative design supports concurrent suppression of pro-inflammatory signaling (for example, TNF-α silencing), reinforcement of mucosal repair and reshaping of gut microbial ecology^85^, ^86^. Notably, similar hybrid engineering concepts have also been extended to microbiome-derived exosome systems. Cui *et al*. reported the use of exosomes derived from *Lactobacillus acidophilus* to encapsulate UiO-66-NH₂ metal-organic framework nanoparticles for delivering TNF-α siRNA. This system shows significant colonic targeting ability and affinity for inflammatory cells after oral administration. The hybrid system not only achieves gene silencing of key inflammatory pathways through TNF-α siRNA but also leverages the natural immune-modulatory properties of probiotic exosomes to simultaneously improve gut microbiota composition and metabolic phenotype, resulting in a multi-pathway synergistic therapeutic effect that combines inflammation suppression, mucosal repair, and microbiome reconstruction in a DSS-induced colitis model^87^.

In addition to oral hybrid systems, exosome-material hybrid engineering can also enhance the retention at inflammation sites and amplify therapeutic effects through localized delivery pathways. Nie *et al*. developed a delivery system that encapsulates IL-27-overexpressing MSCs in dopamine methacrylamide (DMA)-modified GelMA bioadhesive microparticles. Through rectal administration, the system firmly adheres to the inflamed colonic mucosal surface, ensuring sustained release of exosomes, thereby significantly inhibiting inflammatory cytokines, restoring tight junction integrity, and accelerating epithelial repair^88^. Such approaches underscore the complementarity between delivery route, material form and biological function in hybrid therapeutic design.

In conclusion, by combining the colonic/immune cell targeting and excellent biocompatibility of natural exosomes with the engineering advantages of synthetic nanomaterials in terms of loading capacity, gastrointestinal environment tolerance, and stimulus-responsive release, a synergistic regulation of inflammation, barrier repair, and microbiome homeostasis has been achieved. Future clinical translation will require the systematic establishment of a quantifiable "structure-distribution-efficacy" correlation model and the development of standardized, scalable preparation and quality control systems.

## 5. Discussion and Perspectives

Accumulating evidence suggests that exosomes have evolved from being regarded merely as mediators of intercellular communication to multifunctional regulatory platforms involved in IBD pathogenesis, diagnosis, and therapeutic intervention. Preclinical studies indicate that exosomes may contribute to the initiation and progression of IBD through coordinated mechanisms, including modulation of the Th17/Treg immune balance, inhibition of NLRP3 inflammasome-mediated pyroptosis, and remodeling of the gut microbiota. In parallel, the stable presence of exosomes in body fluids, together with their cargo of miRNAs, lncRNAs, and proteins, highlights their potential as minimally invasive biomarkers for disease diagnosis and activity monitoring. Advances in engineering strategies have further enabled the development of exosome-based delivery systems with improved targeting of inflamed intestinal tissues and reduced systemic exposure.

Despite these advances, several critical challenges continue to limit the clinical translation of exosome-based strategies in IBD. Pronounced heterogeneity in exosome composition, biological function, and *in vivo* distribution across different sources complicates standardization, efficacy evaluation, and mechanistic interpretation. In addition, robust and scalable platforms for production, purification, quality control, and long-term biosafety assessment remain insufficiently established. Engineering modifications designed to enhance targeting efficiency may also introduce potential immunogenicity and off-target effects. Furthermore, most current evidence is derived from preclinical models, and systematic clinical validation in large, well-characterized cohorts across different IBD subtypes and disease stages remains lacking.

Future research should adopt a more systematic and cautious approach to advance this field. Integrating multi-omics data with systems biology frameworks may help establish quantitative relationships among exosome composition, inflammatory microenvironments, and therapeutic responses, thereby improving diagnostic and predictive performance. At the same time, the development of standardized engineering workflows and reproducible manufacturing strategies will be essential for ensuring consistency and scalability. Moreover, combining exosome-based platforms with existing therapeutic modalities, such as biologics, small-molecule drugs, or microbiota-targeted interventions, may offer opportunities for multi-target and personalized treatment approaches. Overall, while exosomes represent a promising avenue for bridging mechanistic insights and precision medicine in IBD, substantial methodological and translational challenges must be addressed before their full clinical potential can be realized.

## Authorship contribution statement

Zhong-kuo Zhao: Data curation, Investigation, Writing - original draft. Jia-le Ma: Formal analysis, Investigation, Writing - original draft, Methodology. Fei Lu: Methodology, Writing - review & editing. Shou-Jie Wang: Conceptualization, Funding acquisition, Project administration, Writing - review & editing. All authors have read and agreed to the published version of the manuscript.

## Figures and Tables

**Table 1:** Summary of engineered exosome-based therapeutic strategies for inflammatory bowel disease

Engineering strategies	Specific approach	Experimental model	Route of administration	Biological function	Molecular mechanism	Ref
Engineering of exosome-producing cells	Genetic modulation of NLRP3 expression in MSCs	C57BL/6 mice	Intraperitoneal injection	Promote glycolysis and suppress inflammatory responses	Glut1, IL-10↑	57
Engineering of exosome-producing cells	Hypoxic preconditioning of HF-MSCs	C57BL/6J mice	Tail vein injection	Enhance mitophagy, attenuate oxidative stress and inflammation, and improve intestinal barrier function	PI3K/AKT/mTOR↓	58
Drug loading and cargo engineering of exosomes	MTX loading into GDNVs via incubation	C57BL/6J mice	Oral administration	Maintain intestinal macrophage homeostasis	HO-1↑ IL-1β, TNF-α↓	63
Drug loading and cargo engineering of exosomes	Curcumin loading into GDNVs via sonication	C57BL/6 mice	Oral gavage	Regulate serum metabolite levels and gut microbiota	IL-10↑ IL-6, MPO↓	64
Drug loading and cargo engineering of exosomes	Electroporation loading of TNF-α siRNA into milk-derived exosomes	BALB/c mice	Oral gavage	Alleviate inflammatory infiltration and promote intestinal mucosal barrier repair	TNF-α, IL-1β, IL-6↓	65
Chemical modification of exosomes	HA conjugation via lipid-PEG bridging	C57BL/6 mice	Oral administration	Inhibit M1 macrophage polarization and promote epithelial cell regeneration	ZO-1↑ IL-1β, IL-6↓	73
Physical modification of exosomes	Layer-by-layer coating with chitosan/hyaluronic acid	C57BL/6 J female mice	Oral gavage	Repair the intestinal barrier and reduce cell apoptosis	Bcl-2↑ Bax↓	74
Physical modification of exosomes	TAT-mediated loading of VHH and LL37	C57BL/6 mice	Oral administration	Repair the intestinal mucosal barrier and restore the balance of the gastrointestinal microbiota	TNF-α, IL-1β, IL-6↓	75
Hybrid engineering of exosomes with nanomaterials	Reassembly of GELN-derived lipids into LLNs	Lgr5-EGFP-IRES-CreERT2 mice, B6.Cg-Tg(BAT-lacZ)3Picc/J mice	Oral gavage	Induce intestinal stem cell proliferation and promote intestinal tissue remodeling and regeneration	Wnt/β-catenin↑	80
Hybrid engineering of exosomes with nanomaterials	IL-22 mRNA-loaded lipid nanoparticles derived from GDNVs	C57BL/6 female mice	Oral gavage	Regulate intestinal epithelial homeostasis and accelerate wound healing	IL-22↑ TNF-α, IL-1β, IL-6↓	82
Hybrid engineering of exosomes with nanomaterials	GDNV-coated ZIF-8 nanoparticles loaded with TNF-α siRNA	C57BL/6 female mice	Oral administration	Facilitate siRNA escape from lysosomes, repair the intestinal barrier and restore gut microbiota homeostasis	TNF-α, IL-1β, IL-6↓	85
Hybrid engineering of exosomes with nanomaterials	UiO-66-NH₂ nanoparticles with probiotic EV membrane coating	C57BL/6 female mice	Oral gavage	Suppress inflammation, repair mucosa, and reconstruct gut microbiota	IL-10↑ IL-1β, IL-6↓	87
Hybrid engineering of exosomes with nanomaterials	GelMA-based hydrogel encapsulating IL-27-overexpressing MSC-EVs	C57BL/6 mice	Rectal administration (enema)	Suppress inflammation and repair the mucosal barrier	ZO-1↑ TNF-α, IL-6↓	88

## Abbreviations

IBD: inflammatory bowel disease
UC: ulcerative colitis
CD: Crohn's disease
Th17: T helper 17 cells
Treg: T regulatory cells
MSC-Exos: exosomes derived from mesenchymal stem cells
hucMSCs-Exos: exosomes derived from human umbilical mesenchymal stem cells
BMSCs-Exos: exosomes derived from bone marrow mesenchymal stem cells
SCFAs: short-chain fatty acids
LJ-EVs: exosomes derived from *Lactobacillus johnsonii*
ROS: reactive oxygen species
LFA: lateral flow assay
POCT: point-of-care testing
Hy-Exos: exosomes derived from hair follicle mesenchymal stem cells cultured under hypoxic conditions
PDENs: plant-derived exosome-like nanoparticles
MTX: methotrexate
GDNVs: Ginger-derived nanovesicles
LBL: layer-by-layer
HA: hyaluronic acid
Rabex: Red cabbage-derived EVs
NTs: Tea-derived nanovesicles
DMA: dopamine methacrylamide
